# Do Daytime Activity, Mood and Unit Tumult Predict Nighttime Sleep Quality of Long-Term Care Residents?

**DOI:** 10.3390/healthcare10010022

**Published:** 2021-12-23

**Authors:** Murad H. Taani, Christine R. Kovach

**Affiliations:** College of Nursing, University of Wisconsin-Milwaukee, Milwaukee, WI 53211, USA; ckovach@uwm.edu

**Keywords:** dementia, nursing home, emotion

## Abstract

Based on the premise that stressors can have a cumulative effect on people with dementia throughout the day that contributes to negative consequences later in the day, we examined if daytime activity, unit tumult, and mood were associated with sleep quality. A convenience sample of 53 long-term care (LTC) residents participated in this correlational study. Objective sleep quality was measured using actigraphy, and comorbid illness and level of dementia were control variables. Half of the sample had a sleep efficiency that was less than 80% and was awake for more than 90 min at night. Comorbid illness, negative mood at bedtime, and daytime activity level accounted for 26.1% of the variance in total sleep minutes. Census changes and the use of temporary agency staff were associated with poor sleep. Findings suggest daytime activity, mood at bedtime, and unit tumult should be considered when designing and testing interventions to improve sleep quality.

## 1. Introduction

Sleep quality declines in old age and is particularly poor for long-term care (LTC) residents with dementia [1,2,3,4]. Up to 70% of patients in early-stage dementia have sleep disturbances [4]. Poor sleep is often accompanied by negative health outcomes such as irritability, decreased functional status and performance, decreased attention, increased risk of falls, and elevated mortality risk [5,6]. Compromised sleep quality in people with dementia is also associated with severe cognitive and neuropsychiatric symptoms, behavioral symptoms of agitation, aggressiveness and disinhibition, and poor quality of life [4,7]. Although sleep architecture changes with age, sleep quality is more dependent on physical, environmental, and health factors than on age-dependent sleep changes [8]. Thus, poor sleep quality should be explored and not simply viewed as a normal part of aging.

Poor sleep quality in people with dementia has a multifactorial etiology. Pathophysiological changes related to dementia [9], psychiatric disorders, polypharmacy, breathing disorders, and circadian rhythm disruption have been identified as factors that negatively influence sleep quality [2]. Factors associated with poor sleep/wake patterns in LTC include minimal daylight exposure, nighttime exposure to intermittent light, limited social interaction, and extended time spent in bed [2,10].

There is no conclusive evidence that any medication for the sleep problems of people with dementia is effective [1,3]. Drugs that promote sleep can also lead to negative side-effects for people with dementia, such as increased risk of falls and disorientation [1,3]. As a result, it is imperative to develop non-pharmacological interventions that are feasible for prevention and first line sleep management strategies for people with dementia [11]. While light therapy showed some positive effects on sleep among older adults with dementia [12], enhanced nonpharmacological interventions should be developed, tested, and combined with environmental interventions when feasible [13]. The development of non-pharmacological interventions should be informed by a clear and comprehensive understanding of poor sleep and associated factors.

This study is one part of a larger investigation of factors associated with sleep quality in LTC residents with dementia. The sources of physical pain associated with poor sleep quality were also investigated and are being analyzed by other team members. The variables investigated in this study were identified a priori by an Advisory Committee that included interdisciplinary staff, two physicians, and two community members. Members of the Advisory Committee concluded that daytime activity, mood at bedtime, and factors that could create more tumult on the unit, especially during the daytime, might influence sleep and have not been thoroughly studied.

Based on the Progressively Lowered Stress Threshold Model (PLST) [14], people with dementia have a lowered stress threshold and are less able to tolerate environmental stressors. Environmental demands and stressors that exceed the individual’s stress threshold can contribute to multiple changes in the individual’s daily functioning and behavior, such as reduced social interaction, anxiety, and nighttime sleep disturbance [15,16,17,18]. Most census changes occur during the daytime, and when staffing is inadequate or staff are unfamiliar with procedures, the tumult on the unit escalates. Furthermore, when staff members are not familiar to residents, the care provided may be more stressful and anxiety-producing. There may also be less opportunity to engage residents in physical or social activity when the daytime staffing level is inadequate. This may influence sleep as daytime activity has been suggested to reduce behavioral symptoms and, ultimately, improve sleep [15]. All of these factors were hypothesized by the Advisory Committee to contribute to cumulative negative mood at bedtime and poor sleep.

This study takes one step toward examining the relationship of daytime activity and psychosocial and environmental factors with the sleep quality of LTC residents with dementia. Our scientific premise was that daytime activity and mood at bedtime would impact sleep quality, and that what happened during the daytime would impact sleep. The primary hypothesis was as follows: Controlling for physical illness, daytime activity and mood at bedtime would contribute to nighttime sleep quality for LTC residents with dementia. For a subgroup of people residing on skilled nursing units, we were also interested in examining if there was a difference in sleep quality during the night based on factors that could create more tumult on the unit during the daytime, such as staffing level, use of temporary agency-affiliated certified nursing assistants (CNAs), or unit census changes.

## 2. Materials and Methods

### 2.1. Design, Setting, and Sample

This study utilized a descriptive cross-sectional design and data were collected at three continuing care retirement communities (CCRCs). The CCRCs provide access to older adults who live independently and those who need almost complete assistance with activities of daily living. Inclusion criteria were a diagnosis of dementia and an age of 55 or older. Excluded from the study were residents without dementia, people admitted for short-term rehabilitation, those with acute illness, and anyone with a movement disorder that would interfere with the measurement of movement.

The study was approved by the Institutional Review Board of the University of Wisconsin Milwaukee and written consent to participate was obtained from the legally authorized signatory. The legally authorized signatory was either the resident or the individual legally authorized to make health care decisions for that resident. Consents were solicited for a total of 150 residents, and 62 residents were consented (41% response). Four residents were not eligible due to movement disorder, two were resistant to keeping the actigraph watch on, two withdrew due to scheduling conflicts, and one resident was dropped due to comorbid illness interfering with actigraphy measurement. The resulting sample size was 53; 47 from skilled units and six from independent apartments who were receiving home health care services.

### 2.2. Measurement

Sleep and Daytime Activity. Actigraphy was used to measure movement during the day and nighttime as a proxy for objective sleep quality and activity. The actigraph is not invasive, can be used in natural living environments, and indirectly measures sleep by detecting the absence of movement. It is recommended that actigraphs be worn for a minimum of three consecutive nights when objective sleep quality is the variable of interest [19]. Micro Mini-Motionlogger^®^ actigraphs were worn on the non-dominant wrist from Monday afternoon until Thursday morning and continuously measured physical movement in 1-min epochs (Ambulatory Monitoring Inc., Ardsley, NY, USA). The actigraphs also contain a button called an “event marker” that was pushed at the time of waking and at the time of going to bed for sleep at night.

Sleep logs and event markers were used to record the time the resident went to bed for nighttime sleep and the time the person awakened to start the day. Sleep logs and event markers were completed by certified nursing assistants for residents in skilled care and by the resident, family member, or home health aide for those living in independent apartments. Both the sleep logs and event markers on the actigraph were used to divide the actigraphy output into day and night segments. The wrist actigraph has three data modes available for calculating activity and rest. The proportional integration mode (PIM) calculates the area under the curve for each epoch and is the preferred mode for identifying movement [20]. The zero crossing mode (ZCM) counts the number of times per epoch that the signal crosses a threshold that is set very close to zero and is the preferred mode for capturing lower frequencies, such as those during sleep and rest [21]. Time above threshold counts the amount of time per epoch that the signal is above a set threshold and has multiple limitations for measuring both activity and rest [18]. In this study, the zero crossing mode (ZCM) was used to analyze sleep and the proportional integration mode (PIM) was used to analyze daytime activity outputs for better estimates of movement intensity, daytime activity, movement acceleration, and amplitude [1,22,23].

Objective nighttime sleep quality was the outcome variable and included measures of total sleep time, sleep efficiency, wake after sleep onset, and sleep latency. Total sleep time is minutes of nighttime sleep and sleep efficiency is the proportion of time spent sleeping [24,25]. Wake after sleep onset refers to the total minutes the person is awake during the nighttime from the onset of sleep to the final awakening [25]. Sleep latency is the number of minutes it takes to fall asleep as measured by the first period of persistent inactivity [25].

Predictor variables measured by actigraphy were daytime activity and time asleep. Daytime activity refers to the magnitude of activity on a per-epoch basis during the daytime, as determined by dividing the total-activity score by the number of epochs. Daytime sleep refers to the minutes of the daytime with persistent inactivity.

Mood. Since people with dementia have difficulty articulating their mood, affective states have been reliably observed by trained coders based on facial expression, behavior, and verbalizations [26]. The Observed Emotion Rating Scale rates two positive emotions (pleasure and general alertness) with a possible range of 1 to 5, three negative emotions (anger, anxiety or fear, and sadness) with a possible range of 1 to 5, and higher scores indicating a longer duration of expression of that emotion [27]. The tool is designed specifically for nursing home residents with dementia, and convergent and divergent validity have been established [26]. One trained data collector collected this observational data. The data collector was trained by an experienced researcher in observational measurement of affect and behavior in people with dementia. Videotapes of people with dementia being interviewed were used for training and initial interrater reliability testing. This was followed by observations of consenting residents, which yielded interrater reliability consistently at or above 85%.

Unit Tumult. Unit tumult was conceptually defined as events in the residents’ living area that are deviations from the typical day. Unit tumult data were only available for the participants residing on skilled nursing unit (n = 47). Nominal variables that were reflections of unit tumult were census changes, being cared for by a certified nursing assistant (CNA) from a temporary staffing agency, and a lower than usual staffing level. A census change occurred when there was an admission, discharge, transfer, or death of a resident from that unit during the day or evening shift of the measurement day. Likewise, being cared for by a certified nursing assistant (CNA) from a temporary staffing agency was measured for the day or evening shift of the measurement day. The two facilities in the study that provided skilled care had the highest quality rating for staffing as measured by the Centers for Medicare and Medicaid [28]. Staffing level was coded as normal/above or below, based on the units’ expected day and evening staffing levels for the day and evening shift of the measurement day.

Other Measures. A diagnosis of dementia was obtained from medical record review. Level of dementia was measured with the 30-point Mini Mental Status Exam (MMSE) [29]. A score greater than or equal to 24 points indicates normal cognition, 19–23 indicates mild cognitive impairment, 10–18 indicates moderate impairment, and severe is less than or equal to 9. Comorbid conditions were measured by record review using the Cumulative Illness Rating Scale-Geriatric (CIRS-G) [30]. The CIRS-G quantifies the burden of disease in older adults by rating 13 relatively independent areas grouped under body systems on a 5-point severity scale, ranging from “none” to “extremely severe”.

### 2.3. Procedures

All study data were collected by a trained geriatric nurse research assistant. Following consent, the resident record was reviewed for exclusion criteria, demographic data, and comorbid conditions. The MMSE was administered by the research assistant. Actigraphs were placed on the non-dominant wrist of eligible participants on a Monday and removed on a Thursday. Sleep logs were distributed and the person or persons who would complete the sleep log and press the event markers on the actigraphs were provided with instructions and an opportunity to have questions answered.

For feasibility of data collection, mood and unit tumult measures were collected on one measurement day, chosen by convenience. Mood was scored following a ten-minute observation in the mid-morning and before hora somni (hs) care, i.e., at bedtime. Reports from the certified nursing assistant and/or resident were used to ensure that observations of mood were obtained at least 20 min past the time of any potentially discomfort- or stress-producing event. Census changes were collected from the facility daily census report, staffing level was collected from the scheduler, and use of temporary agency CNA staff for specific residents was collected by having the research assistant ask the CNA caring for the resident on the day and evening shift of the measurement day.

### 2.4. Data Analysis

For descriptive purposes, sleep medians were calculated from the three nights of sleep data collected. For inferential testing, sleep data for the one night that coincided with the day the mood, activity, and unit tumult measures were collected. Hence, if mood and unit tumult variables were collected on a Tuesday, the actigraphy assessments of activity during Tuesday in the daytime and the nighttime measures of sleep that started on Tuesday evening to Wednesday were used for inferential analyses.

Total sleep time is the only measure of sleep quality that had a normal enough distribution for parametric analyses. The variables sleep efficiency, sleep latency, and wake after sleep onset could not be logarithmically transformed and were analyzed as dichotomous variables. Based on norms for sleep efficiency, low sleep efficiency was <0.80 and the higher group was ≥0.80. Based on median splits, we divided sleep latency (<0 and ≥21) and wake after sleep onset (<90 and ≥90) into two groups. Spearman’s rho was used to calculate bivariate relationships between positive mood at bedtime, negative mood at bedtime, daytime activity level, and measures of sleep quality.

Since comorbid illness is known to influence sleep quality, this variable was included as a covariate in the regression model [31]. Daytime activity level and negative mood were related to total sleep time and entered into a hierarchical regression model with comorbid illness as a control variable. Due to a lack of variability in staffing level, this variable was not used in inferential testing. The Cochran’s test of conditional independence determined the independence of sleep quality, unit census change, or agency use dichotomous variables, while controlling for dementia level. Since people with dementia may be less aware of the larger environmental context, dementia level was chosen as a covariate when examining unit tumult [32]. For the Cochran’s test, the level of dementia was dichotomous with an MMSE score of 0 to 17, indicating moderate or severe impairment, and scores of 18 to 23 indicated mild impairment.

## 3. Results

Our sample of 53 LTC residents with dementia (mean age 88.1; SD 9.10) was primarily female (77%), white (98%), and well educated (75% attended college; 23% held a graduate degree).

### 3.1. Daytime Activity and Sleep

The median time awake during the daytime for two days of testing was 9.5 h (4.2 to 16.5) and 9.3 h (0.6 to 14.9). The median time asleep during the daytime was 3.0 h (0 to 9.2) and 2.5 h (0 to 10.5).

### 3.2. Nighttime Sleep Quality

Objective nighttime sleep quality is described in Table 1. There was considerable variability in objective sleep quality. Three residents slept for less than 10 min of one night. Fifty percent of the sample fell asleep in less than 10 min. While the median total sleep time was over 8 h for each night, 41% of the sample had a sleep efficiency that was less than 0.80. Half of the sample was awake after sleep onset for more than 90 min and 25% was awake for more than three hours of the night.

### 3.3. Mood at Morning and Bedtime

Mood was similarly positive in the morning and at bedtime with a median score of 4 and a range of 1 to 5 for both times. Negative mood also had median scores that were the same at both the morning and bedtime measurement times (median = 7, range 3 to 15 at both times). The morning and evening positive mood were correlated (0.472, *p* < 0.001) and the morning and evening negative mood were correlated (0.377, *p* = 0.005).

### 3.4. Factors Predicting Nighttime Sleep Quality

Positive mood during the daytime (total sleep time r = −0.126, *p* = 0.368; sleep efficiency r = −0.004, *p* = 0.978; sleep latency r = 0.006, *p* = 0.966; wake after sleep onset r = −0.048, *p* = 0.735) and at bedtime (total sleep time r = 0.178, *p* = 0.203; sleep efficiency r = 0.108, *p* = 0.452; sleep latency r = 0.178, *p* = 0.203; wake after sleep onset r = −0.027, *p* = 0.850) were not related to any of the objective sleep quality variables.

Negative mood at bedtime was significantly related to total sleep time (r = −0.430, *p* = 0.001), but not to any of the other sleep variables (sleep efficiency r = 0.091, *p* = 0.526; sleep latency r = 0.229, *p* = 0.105; wake after sleep onset r = −0.022, *p* = 0.879). As negative mood increased, the time spent sleeping decreased. Negative mood during the daytime was not related to any of the sleep variables (total sleep time r = −0.158, *p* = 0.260; sleep efficiency r = −0.080, *p* = 0.578; sleep latency r = 0.209, *p* = 0.141; wake after sleep onset r = 0.030, *p* = 0.833).

Daytime activity levels were significantly related to total sleep time (r = −0.461, *p* = 0.001) and sleep latency (r = 0.472, *p* < 0.001), but not to sleep efficiency (r = 0.091, *p* = 0.526) or wake after sleep onset (r = 0.025, *p* = 0.862). People who were more active during the daytime took more time to fall asleep and slept less at night.

Table 2 shows that the variables comorbid illness, negative mood at bedtime, and daytime activity level accounted for 26.1% of the variance in total sleep minutes (*p* = 0.002). When entered into the model alone, comorbid illness was not a significant predictor (*p* = 0.832). At Step two, comorbid illnesses and negative mood at bedtime accounted for 19.3% of the variance, a change of 19.2%, which was statistically significant (*p* = 0.001). Step three of the model showed that 6.8% of the variance in total sleep time was uniquely accounted for by daytime activity levels (*p* = 0.039). Negative mood at bedtime and higher levels of activity were associated with sleeping less.

### 3.5. Unit Tumult and Sleep Quality

The units were exceptionally well staffed with only four shifts of 94 that were short-staffed. Of the 47 participants on skilled units, 23 were cared for by a temporary agency CNA for 31 shifts of 94 (33%) measured.

Controlling for level of dementia, total sleep time and sleep efficiency were related to census changes (Cochran’s = 4.221, *p* = 0.040 and Cochran’s = 4.660, *p* = 0.031). Table 3 compares total sleep times between participants who had a unit census change and those who did not. The largest subgroup sleeping more than 7 h a night (n = 17, 36%) were those who did not have a census change. Sleep latency (Cochran’s = 0.004, *p* = 0.949) and wake after sleep onset (Cochran’s = 0.381, *p* = 0.537) were not related to census changes.

Controlling for level of dementia, the total sleep time was related to the use of agency CNAs (Cochran’s = 5.192, *p* = 0.023). Sleep efficiency, sleep latency, and wake after sleep onset were not related to the use of CNA agency staff (Cochran’s = 0.358, 2.414, and 0.103; *p* = 0.549, 0.120, and 0.748, respectively).

## 4. Discussion

This study showed that nearly half of the participants had a sleep efficiency that was less than 80% and were awake for more than 90 min at night, which suggests that poor sleep quality should be considered an important health concern for this group. These findings are consistent with other studies that reported poor sleep among LTC residents with dementia [33,34,35]. To further evaluate the relationship of daytime activity, mood, and unit tumult to objective nighttime sleep quality of LTC residents with dementia, we observed that the factors influencing total sleep time included comorbid illness, negative mood at bedtime, and daytime activity level. We also found that census changes influence total sleep time and sleep efficiency, while the use of temporary agency influences total sleep time.

Daytime activity levels were significantly related to total sleep time and sleep latency, but not to sleep efficiency or wake after sleep onset, and those who were more active during the daytime took more time to fall asleep and slept less at night. These findings could indicate that the sample who was more active was also healthier in general, and therefore did not spend as much time in bed in a state of inactivity. Current evidence of the relationship between physical activity and sleep remains inconsistent. While some studies demonstrated that objective sleep quality of nursing home residents with dementia was significantly improved by being physically active [36,37,38,39], other studies did not find a relationship [13,40]. Possible explanations for these mixed results are the differences in the individuals and environments, as well as a lack of consistency of sleep and activity measures across studies. For example, one study conducted among community-dwelling older adults with Alzheimer’s disease did not reveal a relationship between walking, total wake time at night, and Sleep Disorders Inventory score [40].

Our findings showed that positive mood during the daytime and at bedtime were not related to any of the sleep variables. The effect of positive mood states on sleep in older adults with dementia gained less attention in the literature [41]. One study showed that healthy individuals high in the tendency to experience and exhibit positive affect were found to report higher levels of sleep quality relative to individuals low in dispositional positive affectivity [42]. Another study showed that healthy individuals who were asked to concentrate on happy thoughts, listen to cheerful music, and develop an intense positive mood exhibited shorter sleep onset latency relative to baseline, which indicates they were able to benefit from a positive affective state before sleep [43]. Future research is needed to confirm our findings and further explore the role of positive mood as a possible protective factor for sleep disturbance in LTC residents with dementia.

Negative mood at bedtime was significantly related to total sleep time, although negative mood during the daytime was not related to any of the sleep variables in our study. We observed that as negative mood at bedtime increased, the time spent sleeping decreased. The main manifestations of mood disorders included sadness, depressed mood, and anxiety, and could worsen sleep disturbance and lead to poor sleep in older adults [44]. Individuals with depressed mood often exhibit abnormal sleep–wake cycles and reduced circadian amplitudes in daily activity and other physiological functions [45]. A growing body of literature suggests that sleep and mood states are associated through a bidirectional relationship. Mood states may lead to poor sleep and sleep disturbance may negatively affect emotional well-being [41]. However, while experimental research has addressed the negative effects of sleep variations on emotions, very few studies examined the effects of manipulated emotions on sleep. The effect of mood states on sleep and its underlying mechanism should be elucidated by future research [41,46].

We further found that total sleep time and sleep efficiency were related to census changes. More participants slept longer when there was not a census change and high sleep efficiency was more common when there were no census changes than when there were census changes. Census changes are usually associated with admissions, discharges, and transfers, which are all well-known to be high-activity, time-demanding processes and associated with a noisy, crowded, or chaotic environment in many healthcare settings. Similar results have been found in a study indicating that the timing of staff shift patterns and institutional regimes, as well as routines such as the noise of squeaking medication carts or pill crushing, negatively impact sleep [47]. Other studies demonstrated a relationship between noise and sleep [48,49]. These studies reported that sleep could be further disrupted by unacceptable levels of noise and light in LTC and that sleep was markedly impaired in an environment of elevated sound and light levels in acute care settings. It is worthy to note that issues with circadian rhythms are considered an important mechanism responsible for poor sleep [50] and have not been investigated in our study. Future research should consider the role of noise, circadian rhythms, and light on sleep in people with dementia, which can help nurses and other clinicians to initiate sleep improvement protocols. The findings also showed that more participants slept longer when they were cared for by their consistent CNA compared to the number who slept well and had an agency CNA. We did not find a relationship between sleep efficiency, sleep latency, and wake after sleep onset and the use of consistent CNA staff. Although studies on the relationship between consistent staff assignment and sleep quality is limited, having consistent staff assignments who know the residents’ needs very well and can recognize early signs of distress is a key component in any dementia care approach and can decrease the staff frustration as well [51,52].

Findings from this study were mixed, but did uncover some significant relationships that are consistent with the person–environment interactions postulated in the Progressively Lowered Stress Threshold model. Taken together, as the negative sequelae of sedative and sleep medications can be devastating, this study supports considering daytime activity, mood, and unit tumult as factors that influence sleep among LTC residents with dementia. Such factors should be considered when designing and testing non-pharmacological interventions to improve sleep among this population. Efforts to improve sleep may not only improve the sleep quality and quality of life among the LTC residents with dementia, in general, but may also substantially reduce the staff and caregiver burden [53].

This study has several limitations. The sample size was determined by the number providing consent and meeting eligibility criteria, and the small sample size might be viewed as a limitation. Utilizing a convenient sample may have added to the sampling error. Since we did not have permission from the IRB to collect information on those who did not provide consent to participate, we do not know if the group of consenters was substantially different from the non-consenters. If differences were present, there could be a potential bias due to self-selection and external validity of the study could be compromised. Collecting mood and unit tumult measures on only one day and on a day that was chosen by convenience could have introduced measurement error and bias. Variations in who recorded sleep log information could have introduced error. The use of multiple bivariate analyses and the dichotomization of the participants’ scores could have influenced results and increased the likelihood of Type I error. Multiple variables, including medications that can influence sleep, were not controlled in this study. The dichotomization of skewed data, inaccuracy of actigraphy in detecting and distinguishing sleep versus activity, and potential differences across units might also be considered limitations. Nonetheless, the relationships identified between daytime activity, mood, unit tumult, and sleep provide a foundation for future studies that can explore causal mechanisms.

## 5. Conclusions

Research on sleep among LTC residents with dementia is sparse. Our findings extend previous knowledge on sleep among LTC residents with dementia by demonstrating that negative mood, census changes, and inconsistent staff assignment were associated with poor sleep. Although the studies are inconsistent on the relationship between daytime activity and sleep, our results showed that daytime activity was associated with low total sleep time and increased time to fall asleep. Despite the limitations of this study, it is still an important step in presenting the factors influencing sleep and providing insights for future studies on improving sleep among LTC residents with dementia. While research on sleep in this population is still needed, we advise that all factors that influence sleep presented in this study and other studies should be given adequate consideration when caring for LTC residents with dementia. Addressing nursing practices and creating a multidisciplinary approach to address the factors that influence sleep could result in modifications to improve sleep without compromising the quality of care.

## Figures and Tables

**Table 1:** Description of Nighttime Sleep Quality over 3 days (N = 53).

Day and Sleep Measure *	Median	Minimum	Maximum
Total Sleep Time			
Day 1	507	1	804
Day 2	500	9	825
Day 3	516	1	773
Sleep Latency			
Day 1	7	0	96
Day 2	9.5	0	633
Day 3	7.5	0	780
Sleep Efficiency			
Day 1	84	40	100
Day 2	85	44	99
Day 3	83	36	99
Wake After Sleep Onset			
Day 1	93	0	451
Day 2	92	5	363
Day 3	100	0	679

* Total sleep time, sleep latency, and wake after sleep onset are expressed in minutes. Sleep efficiency is expressed as a percentage with cutoffs of sleep efficiency at below 80% and 80% or higher, serving as clinically meaningful cutoffs for “poor” and “good” sleep efficiency.

**Table 2:** Summary of Hierarchical Regression Analysis with Total Sleep Minutes as Criterion (N = 53).

Step and Predictor Variables	R^2^	ΔR^2^	β	t	*p*
Step 1					
Comorbid Illness	0.001	0.001	0.030	0.213	0.832
Step 2					
Comorbid Illness			0.093	0.725	0.472
Negative Mood at Bedtime	0.193	0.192 *	−0.443 *	−3.451	0.001 *
Step 3					
Comorbid Illness			0.016	0.125	0.901
Negative Mood at Bedtime			−0.340 *	−2.548 *	0.014 *
Daytime Activity	0.261	0.068 *	−0.287	−2.121 *	0.039 *
F(3, 52) = 5.776, *p* = 0.002					

* Statistically significant *p* < 0.05.

**Table 3:** Total sleep time and census change.

	No Census Change	Census Change
Total sleep time (≤7 h)	10	5
Total sleep time (>7 h)	17	15

## Data Availability

The data presented are included in this study; additional data may be provided by the corresponding author on request.
